# Concepts of self in dementia research: towards theoretical integration

**DOI:** 10.1007/s11019-025-10253-y

**Published:** 2025-02-14

**Authors:** T. J. van Woerkum-Rooker

**Affiliations:** Faculty of Philosophy, Theology and Religious Studies, Radboud University, Nijmegen, Gelderland The Netherlands

**Keywords:** Dementia, Alzheimer’s disease, Self, Conceptualizations of self, Selves

## Abstract

Research on understanding the self of persons with dementia (PWD) has increased significantly in the past decades across various fields of research. This has led to a profusion of novel conceptualizations of self. Meanwhile, the rise in dementia diagnoses worldwide presents us with complex global societal and individual challenges. Since the understanding of the self of PWD is vital for improving their well-being, autonomy and care needs, this article argues that there is a need to integrate and systematize these conceptualizations of self. The current state of conceptual unclarity undermines the wellbeing of PWD, since it impedes the exchange and development of (empirical) research results and ideas. With the aim of uniting and systematizing the conceptualizations of self in research on PWD, in order to develop a pragmatic, clustered approach based on the research of the field itself which can be applied in an empirical setting with PWD, this article departs from the literature reviews from the various fields involved in the research on the self of PWD. By focusing on the theoretical overlap between the conceptualizations of self employed in these reviews, four overarching clusters of self-aspects can be formulated: minimal, embodied-embedded, reflective and socially-embedded self-aspects. These clusters jointly provide the ground for self-continuity in PWD. This clustered approach provides a framework which unites the current field of research, within which new findings can be integrated and which can be applied in an empirical setting.

## Introduction

The latest estimate from the World Health Organizations suggests that over 55 million people worldwide are currently living with dementia (WHO 2023). This number is anticipated to increase by nearly 10 million new diagnoses annually. These figures present significant challenges on a societal and individual level. Each number represents an individual who has to adapt to serious cognitive decline and is in need of specialized care. The influence of dementia on a person’s life is far-reaching and brings about changes that significantly impact facets we often perceive as integral to our ‘self’, like our memories, our social interactions, the ways in which we express ourselves and make our way in the world.^1^

Research on the ways in which dementia impacts the self has increased over the past years. Whereas the prevailing understanding used to be that the self was largely or entirely lost during the dementia process, new developments in research spanning diverse fields have demonstrated that this belief is oversimplified (Caddell and Clare 2010). These new findings indicate that dementia impacts the self in various ways, but without entirely eradicating it. This more nuanced understanding of the self of persons with dementia (PWD)—as affected by dementia but persisting in various forms throughout the illness—has, however, led to a problem. It has generated a profusion of novel conceptualizations of self, often accompanied by various methodological frameworks, that complicates joint theory-building in (empirical) research. This, in turn, has led to difficulties integrating theoretical insights derived from these research outcomes into the dementia care practice.

Therefore, the growing impact of dementia on our societies and the crucial role of our understanding of the self for the wellbeing and autonomy of individuals living with a dementia diagnosis demands a moment of reflection on the current field of research. In this article, it is argued that there is a need for an alternative approach, since the current field of research on the self of PWD has become a complex tangle of concepts and frameworks, which is nearly impossible to navigate theoretically and to apply in empirical research. This is especially urgent given that, as Van Wijngaarden, Alma and The (2019), argue: ‘Although most health professionals and health researchers are profoundly aware that good care not only requires interventions, therapies and/or treatment, but also implies that professionals foster attunement to how an individual experiences things, the number of robust studies that contribute to a deeper understanding of the nature and meaning of living with dementia is still limited. Experiences of living with dementia from the perspective of people with dementia have been explored in a number of valuable studies, but, again, the results focus mainly on psychological impact and coping strategies’ (p. 3).

Therefore, in order to contribute to viable, interdisciplinary empirical research on the self of PWD that can contribute to the improvement of dementia care practices, this article has the following aim: to integrate and systematize the current multiplicity of conceptualizations of self in individuals with dementia, in order to develop a pragmatic, clustered approach based on the research of the field itself which can be applied in an empirical setting with PWD.

It is important to note that this clustered approach is by no means an attempt to formulate an encompassing notion on what *the* self (of PWD) *is.* It is a way of delineating and distinguishing aspects of the self that can be used in empirical research based on the research results of the various fields involved. This entails doing justice to the (theoretical) richness of the concepts involved whilst at the same time taking their clinical relevance into account. Therefore, in line with the aim of the article, some theoretical philosophical nuances and intricacies surrounding these various conceptualizations of self across the various fields are beyond the scope of this current article.

We will proceed as follows. Firstly, the method used for a narrative literature review is elaborated on (“Method” section), including the eligibility criteria (“Eligibility criteria” section), the literature search (“Literature search” section), the study selection (“Study selection” section) and data collection (“Data collection” section). Following this, the reasons why the field of research has become problematic are illustrated (“From the unitary to the multidimensional self” section), before proposing a clustered approach following from the existing literature itself (“Towards theoretical integration” section), consisting of minimal self-aspects (“Minimal self-aspects” section), embodied-embedded self-aspects (“Embodied-embedded self-aspects” section), reflective self-aspects (“Reflective self-aspects” section) and socially-embedded self-aspects (“Socially-embedded self-aspects” section). Finally, conclusions are drawn on how we should understand the self of PWD based on this clustered approach and its implications for future research (“Conclusion and discussion” section).

## Method

Since research on the self in dementia consists of various research fields (psychology, neurosciences, philosophy, social sciences etc.), the decision was made to focus on review articles from these various fields.^2^ This way, as much research ground as possible could be covered, since these reviews already represent the ongoing research in their specific field. This would enable an overarching analysis, covering all these different fields.

Regarding the type of analysis, the decision was made to conduct a narrative literature review, i.e. a non-systematic review, based on these review articles from the various fields. Conducting a narrative literature review facilitates the exploration of a broad spectrum of varied research findings while, at the same time, permitting a subjective evaluation of the literature and the formulation of new research directions (Ferrari 2015; Baumeister and Leary 1997).

## Research question

How can the current multiplicity of conceptualizations of self in research on persons with dementia be integrated and systematized, to be used in an empirical setting?

### Eligibility criteria

Review articles published between 01-01-2000 and 07-11-2023 (day of search) were included. Review articles outside this timeframe were excluded. All other types of articles besides reviews were also excluded. Only review articles that focus on adults diagnosed with a type of dementia were included. No specifications were formulated regarding the type of dementia. Review articles focusing on other types of cognitive impairments (such as Korsakoff or Mild Cognitive Impairment) were excluded. To be included, the focus of the review articles had to be on the self of the individual diagnosed with dementia. Accordingly, review articles focusing on the perspective of others surrounding the individual with dementia (such as relatives, health care professionals or (in)formal caregivers) were excluded. Besides focusing on the individuals with dementia themselves, the review articles also had to focus on conceptualizations of self and possible related self-aspects to be included. Review articles addressing related topics to conceptualizations of self/self-understanding (such as quality of life, wellbeing, coping) or related (practical) implications (such as intervention methods, assessment/diagnosis tools, treatments/therapies, research instruments) were excluded. Only review articles completely written in English (title, abstract, full text) were included.

### Literature search

The search was conducted on November 7th, 2023. Together with an experienced librarian of Radboud University, the author created a search strategy focused on review articles. In doing so, they balanced between, on the one hand, identifying all relevant reviews and, on the other hand, navigating the vast amount of literature concerning the self, dementia and related topics (for example, the word ‘self’ is often merged with other words, such as ‘self-registered’. This initially resulted in hits related to, for instance, ‘self-registered food intake in dementia’ which are not relevant to the current article).

The decision was therefore made to focus on references that had both dementia (or Alzheimer’s disease) *and* self (or selves) in the title, combined with three key words that identify the article type (review). This resulted in a focused search, identifying reviews on the understanding of the self in dementia. Four databases were searched: PubMed, PsycINFO, CINAHL and Web of Science. The same search string was adjusted to the terminology used by the specific database. Additionally, the first 10 pages of Google Scholar were searched to make sure no important review articles were missed, but no additional review articles were found. An example of one the search strategies used (for PubMed) is: (Alzheimer*[Ti] OR Dementia*[ti]) AND (self*[ti] OR selves[ti]) AND (review[pt] OR systematic review[sb] OR review[tiab]).

### Study selection

All results were imported into EndNote 21. The search resulted in 232 articles. After removing all the duplicates, 105 unique references remained. The selection that followed was done based on the eligibility criteria for inclusion and exclusion as mentioned in ”Eligibility criteria” section. This process resulted in 14 review articles from which the data could be extracted for the narrative analysis. One of the reviews could not be accessed online. The author of this article was contacted by the author via e-mail, but did not reply and was therefore excluded in the process. Ultimately, 13 review articles remained (see Table 1).

**Table 1:**
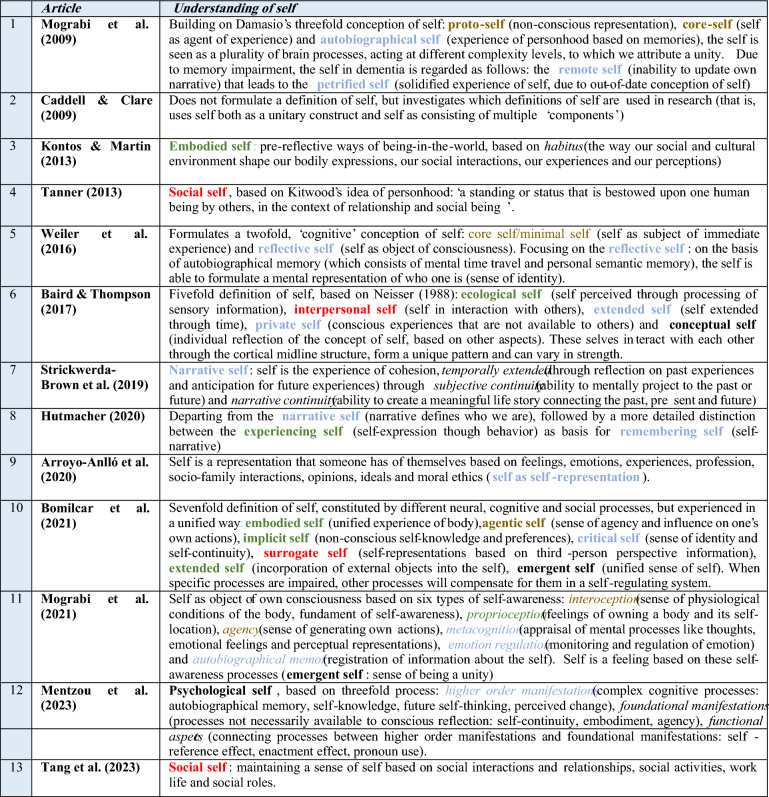
Overview of the ways in which the self is understood in the reviews on the self in dementia. Articles are chronologically displayed based on the month of publication. The notions have been categorized based on their cluster (brown indicates a minimal self-aspect cluster, green indicates an embodied-embedded self-aspect cluster, blue indicates a reflective self-aspect cluster and red indicates a socially-embedded self-aspect cluster. In the review literature, several umbrella terms are used to indicate the experienced sense of unity arising from the interaction between the various self-aspects. Since these umbrella terms themselves are empty concepts without the self-aspects they consist of, only the self-aspects they consist of have been clustered in the text and categorized in colors in Table 1.The four umbrella terms are: ‘psychological self’ (Mentzou et al. 2023), ‘emergent self’ (Bomilcar et al. 2021; Mograbi et al. 2021) and ‘conceptual self’ (Baird and Thompson 2018)

### Data collection

Data collection, appraisal and synthesis was carried out by the author, continually provided with feedback from the research team, consisting of two philosophers of mind, two empirical researchers specialized in (health)care research and a clinical geriatrician. Data collection, appraisal and synthesis was done in an inductive way, by analyzing the concepts of self that emerged from the 13 review articles overarching clusters were formed. To connect the various research results the precise meaning sometimes needed to be interpreted, because of the varied (and sometimes ambiguous) usage of terminology in several review articles. For example, concepts like (self-)consciousness, (self-)awareness, personhood, identity, self(hood) are often used interchangeably or without an explicit statement on the precise meaning, relation between concepts or mentioning multiple interpretations.

## The self in dementia research

### From the unitary to the multidimensional self

The concept of ‘self’ is notoriously hard to define. Many researchers from various fields have tried to find encompassing notions of what we exactly try to describe when we use the word ‘self’. This is also the case in the field of dementia research; a field consisting of many disciplines, each with their own research methodologies, frameworks and reference points, all developing their own conceptions of the ways in which the self is impacted by dementia. In their well-known and often referenced review article, Caddell and Clare (2010) were among the first to address the problem of the disparate body of conceptualizations of self utilized in dementia research. They highlighted the need for more uniform concepts, research methods and interdisciplinary theory-formation. However, since the publication of this review, the number of conceptualizations of self and often accompanying frameworks in research on the self in dementia has increased considerably. This multitude is demonstrated in the overview of the current body of review literature on the self in dementia (see Table 1). Table 1 shows the wide range of concepts of self (in bold, colored) and multiple other related aspects of certain concepts of self (in italics), as matching the criteria of the literature search (see “Eligibility criteria”) section.

It is important to note that Table 1 reflects a significant shift in the understanding of the self, from a unitary perspective towards a multidimensional perspective. Although a somewhat ambiguous concept on its own, a unitary notion of self is often understood as a type of ‘core’—often based on reflective abilities—that remains unchanged throughout one’s life and that is integral to being able to talk about the existence (or lack) of someone’s self. This ‘core’ understanding of self is the product of centuries of Western intellectual history in which the self has primarily been defined in terms of reflective abilities.

This is apparent in Locke’s, to this day still, influential notion of personal identity. Locke was influenced by Descartes understanding of selves as ‘thinking things’. Descartes argued that the self is constituted by rational thought (such as the ability to doubt, reflect and reason), distinct from and non-reducible to the material body. This idea contributed to Locke defining a ‘person’ as “a thinking intelligent Being, that has reason and reflection, and can consider it self as itself, the same thinking thing in different times and places” (Locke 1690/1975*,* Bk. 2, Ch. 17, § 9). The latter part of Locke’s definition is sometimes referred to as ‘reidentification’ (the ability to consider yourself the same person throughout time by yourself and others), but in what follows the terms ‘self-continuity’ and ‘continuity of self’ will be used.

This long tradition of focusing on reflective abilities as solely constitutive of the self and self-continuity (i.e., ‘the unitary notion of the self’) has not been confined to the academic domain. Research has shown that this understanding has led to a widespread folk-notion of the self, in which people intuitively connect the loss of reflective abilities to the loss of selfhood (Nichols and Bruno 2010). Accordingly, this view has had great impact on how the self is perceived in the context of dementia, precisely because dementia often impacts reflective abilities, resulting in the belief that living with dementia would ultimately lead to a loss of self. After all, this view implies that without reflective abilities there is no ground for the continuity of self (Cohen and Eisendorf 2001).

However, in dementia research, including all the reviews as mentioned in Table 1, a shift towards a more multidimensional way of thinking about the self is noticeable. This development is not exclusive to dementia research, but apparent in all fields of research related to the understanding of the self. In this line of thought, the self is not ultimately dependent on a specific (reflective) core, but is constituted by several reflective and pre-reflective aspects (Gallagher 2013). Self-continuity, therefore, is no longer solely based on reflective abilities (such as formulated by Locke), but follows from both reflective and pre-reflective abilities in various ways, for example through embodiment or social interaction. Following from this multidimensional understanding of self, impairment due to dementia in one or several of these aspects is no longer seen as (leading to) a loss of self, but as a ‘change’ in the interaction between the several aspects that shape the (continuity of) self. In this interaction, some aspects may become more or less prominent than others during the course of dementia. However, concerning the question which aspects constitute the self in dementia, the multitude of conceptualizations of self as presented in Table 1 demonstrates that this can’t be answered unequivocally.

### Towards theoretical integration

The shift towards a multidimensional perspective in the research on the self of PWD has, perhaps inevitably, been followed by the development of an array of conceptualizations of self (see Table 1). The (main) aim of most of the review articles shown in Table 1—eleven out of thirteen—is to contribute to the dementia care practice (see Table 2 in Appendix). However, one could question the utility of this array of conceptualizations of self and aspects in understanding the impact of dementia on the self. This multitude of conceptual variations of self could potentially hinder rather than aid the advancement of dementia care, since it has resulted in a complex theoretical tangle of concepts and frameworks.

Yet, even though the review literature seems to show a multitude of distinct concepts at first glance, when taking a closer look, these concepts actually contain a lot of overlap. This raises the question whether the number of concepts could and should be reduced to create order in the current state of conceptual chaos. Not only would this provide conceptual clarity, it would also enable the establishment of cross connections between existing and future research results. This all, in turn, would also contribute to the improved translation and implementation of theoretical insights into dementia care practices.

That being said, it is important to note that this clustering would result in a pragmatic notion of the self, which should not be understood as a philosophical claim on which aspects precisely constitute the self or an answer to the question what *the* self *is*. In what follows, the various notions of self as presented in Table 1 will be compared and mapped, thereby showing that that there is reason to validate a four-part clustering of (1) minimal, (2) embodied-embedded, (3) reflective and (4) socially-embedded aspects of self. This will be achieved in an inductive way—i.e. letting the clusters follow from the review literature itself instead of departing from a preconceived framework – thereby covering all the conceptualizations of self and related aspects used. Although, undoubtedly, based on the current research, different clusters are possible as well, there needs to be a balance between theoretical depth and practical applicability. These four clusters will in “Clustering conceptualizations of the self in dementia research” section be discussed in more detail and shown how they follow from the existing review literature.

### The need for a new clustered approach

Within research on the self, others have similarly proposed to cluster self-aspects (e.g. Neisser 1988; Harré 1997; Damasio 1999). Therefore, one might wonder how the current clustered proposal relates to, and differs from, those existing accounts.^3^ To be clear, I acknowledge the importance of these existing accounts. In some ways the proposed clustered approach of this article builds on them, if only because the review articles that feed into the clustering also rely on some of those existing accounts. However, there are three reasons for why a novel clustering is required.

First, the aforementioned accounts have been developed in and for specific contexts (e.g. neuroscience in the case of Damasio, cognitive psychology in the case of Neisser, and cultural and social psychology in the case of Harré). However, the context of dementia (care) implies that other fields of research (that are left out by e.g. Damasio and Neisser) need to be included, such as health sciences (e.g. Tang et al. (2023)) and social sciences (e.g. Tanner (2013)). The complexity of current day dementia care requires an interdisciplinary approach, since both extramural and intramural care depend on the collaboration of various healthcare professionals (nurses, psychologists, doctors, social workers, and so on) from whom the majority of is informed by various and different fields of research. This reinforces the need for a new clustered approach that crosses disciplinary boundaries and that follows from these various fields of research involved, in order to be truly informed and effective.

Secondly, these aforementioned accounts all precede the current proliferation of conceptualizations of self within dementia research, dating back to around the turn of the twentieth century. Like other fields of research working on understanding the self, dementia research has only relatively recently made the shift from approaching the self in a multidimensional way, encompassing both reflective and pre-reflective aspects of self, instead of the previously prevailing unitary understanding of self. Therefore, the current proliferation of conceptualizations of self requires a new approach that allows for these new research results and approaches to be integrated. In this, it is vital that such an integration of research results in an overarching clustering—like the clustering proposed in this article—follows from these various fields involved themselves, instead of applying an already existing framework to new results. Applying these already existing frameworks might not be fully sufficient to cover the insights that have emerged (since the initial development of those aforementioned frameworks). Dementia research itself should be the basis for any clustering of research, instead of it having to fit within a certain preconceived framework.

Thirdly, the clustered approach presented in this article offers a middle ground between coarse grained and fine grained approaches to the self in dementia. Research on the self in dementia has diverged into roughly two strands, where some researchers work on coarse grained perspectives that focus on understanding one particular aspect of self (often reflective aspects), while other researchers work on very fine grained perspectives, developing ever more detailed understandings of (sub-)aspects of self. This has also contributed to the current state of conceptual chaos. However, in view of the expected (exponential) increase in PWD and the challenges intramural, extramural and informal/family ‘at-home’ care providers are already and will increasingly be facing, such a middle ground—which can be applied empirically and eventually within caregiving contexts—is urgently needed. Aiming for a middle ground will inevitably imply some loss of theoretical nuance, but enable us to bridge the gaps between academic research, the dementia care practice and the everyday lives of PWD. It will also allow for future empirical research from the perspective of the PWD themselves. In this, the presented clustered approach aligns with the aim of this article, of contributing to viable, interdisciplinary empirical research that can add to the improvement of dementia care practices.

## Clustering conceptualizations of the self in dementia research

In what follows, the pragmatic fourfold clustered approach consisting of (1) minimal self-aspects, (2) embodied-embedded self-aspects, (3) reflective self-aspects and (4) socially-embedded self-aspects will be described in detail, and in doing so, shown how it follows from and aligns with the findings from the current body of review literature on the self in dementia**.**

### Minimal self-aspects

The idea of minimal self is often used to describe the pre-reflective aspects of self that enable us to have experiences as subjects. Zahavi describes the minimal self as ‘a first-person givenness’ of experience, thoughts and feelings (Zahavi 2005, p. 124). By this, he means that every experience always implicitly implies that *someone* is experiencing it, from a unique point of view—hence, always already implicating a very minimal sense of self, also called ‘mineness’. For example, when you are touched by someone, you are immediately aware from your own perspective that *you* are the one who is being touched. The minimal self is often considered to include the sense of ownership of our experiences (that I am the one who is experiencing) and the sense of agency (that I am the one who is the initiator of an action) (Vogeley and Gallagher 2011; Zahavi 2011). This aspect of self is thought of as a given, implied by the structure of experience itself. Hence, ‘mineness’ implies identifying a ‘me’, since experiences implicitly refer to a *self* having the experience. Yet, ‘mineness’ in the sense of having a minimal self, does not necessarily involve self-continuity, as the notion applies first and foremost to experiences in the here and now.^4^

The notion of a minimal self is reflected in several ways in the review literature (Bomilcar et al. 2021; Mograbi et al. 2009, 2021; Weiler et al. 2016). Weiler et al. (2016) briefly touch upon the minimal self by describing the minimal self as a core self that is temporally and spatially confined to the here and now. This notion of a core self, based on the work of Damasio, is seen as being the immediate subject of experience (as in Zahavi’s notion of the minimal self). Mograbi et al. (2009) also refer to Damasio, concerning the notion of the proto-self. They define the proto-self as ‘a non-conscious representation of the organism’, which can be understood as the information the brain receives from the body about the body, comparable to the notion of interoception (Mograbi et al. 2009, 2021). The proto-self and minimal self are different, as the proto-self is not consciously experienced, while the minimal self is. The former can be seen as the physiological foundation necessary for the latter to emerge. In other words, the minimal self builds on the proto-self’s interoceptive processes, enabling this pre-reflective form of consciousness.^5^

However, although an important and fundamental aspect of understanding the self (of PWD), the minimal self is likely to be of limited significance in an empirical and care giving context. The immediate awareness of yourself as a self depends on processes that remain intact despite cognitive impairment. Put differently, the impact on reflective, social or bodily abilities tends to leave the non-reflective nature of direct experience unaffected. However, the notion of minimal self will not be excluded in this clustered approach aimed at empirical research from the outset.

### Embodied-embedded self-aspects

Embodied and embedded aspects of self are often understood as distinct but intimately connected aspects of self (Cassam 2011). The notion of embodied self refers to the role our bodies play in the way we shape and experience who we are. Having a body enables us to move about in the world, experience all types of sensations and interact with others (for example, through touch and facial expressions). This notion of embodied self is closely linked with the concept of embedded self. Embedded self indicates that we are in constant interaction with our environment and thereby shape ourselves through our surroundings. These surroundings can be our material surroundings (such as the way in which we use technology like smartphones to navigate daily life, or the way in which we express ourselves by how we decorate our home) or our immaterial surroundings, such as the (cultural) ideas and norms we internalize (since this second notion of embedded self is related to social self-aspects, it is elaborated on in “Socially-embedded self-aspects” section). All these interactions with our surroundings happen through our bodies; the fact that we have a body makes it possible for us to have these interactions with our surroundings that shape us. The embodied self offers a foundation for self-continuity: the persistence of your body and your ways of doing things, for example in habits, implies the persistence of certain aspects of your self. The notions of the embodied and embedded aspects of self are reflected in various ways in the review literature, by looking at the body as self-expression, the relatedness between embodiment and embeddedness and the notion of body memory as described below (Baird and Thompson 2018; Bomilcar et al. 2021; Hutmacher 2020; Kontos and Martin 2013).

With regard to the first, the body as self-expression, both Bomilcar et al. (2021) and Kontos and Martin (2013) argue that embodiment can be seen as foundational for the self, since having a body enables us to have experiences, perceptions and (social) interactions in the first place. Embodiment enables (non-verbal) self-expression, for example through gestures, facial expressions, habits, appearance and creativity. This way of self-expression remains a possibility for PWD to express who they are, even when suffering from cognitive impairment like impaired speech. In doing so, embedded self-aspects can be involved, since all kinds of objects can be integrated in embodied self-expression, such as clothing, musical instruments or the tools we use for our hobbies, can play a functional role in acts of self-expression. This interaction between embodiment and embeddedness can be found in the notion of the ‘ecological self’ in the review of Baird and Thompson (2018). In their review on the self in dementia related to music they show how the body remains a way in which PWD can express their selves throughout the course of their illness. They demonstrate this with examples related to music, like the case of Betina, a former piano teacher, for who her retained ability to play a musical instrument remains a way to express aspects of her self.

Hutmacher (2020) approaches these aspects of self in a different way in what he calls the ‘experiencing self’. Hutmacher builds on the notion of body memory by Fuchs (2012, 2018). In his work on body memory, Fuchs argues that our experiences are not only stored in our brain, but also in our bodies. Body memories aren’t ‘passive files’ collecting dust on a shelf, they actively influence our reactions, emotions and expressions in present experiences. Hutmacher (2020) uses the example of a person with dementia who is unable to explain why it is appropriate behavior to lower your voice when entering a church but who nevertheless does lower their voice while being in church and a person with dementia who had to hide in a basement during the second world war and now refuses to enter the basement of the nursing home, without being able to say why. All these responses are based on past experiences, stored in our minds *and* bodies. According to Hutmacher, this shows how our embodied self is extended through time, since memories of past experiences that are ‘filed’ in our body influence our current experiences and behavior. This, according to Hutmacher (2020), shows how PWD, despite possible memory impairment, maintain an experiencing self that ‘is spatially-temporally structured and influenced by the history of the individual’ (p. 162). While PWD might not be able to recall certain memories or express themselves verbally, embodied expression thus provides the ground for self-expression and self-continuity, since these embodied expressions reflect their own personal life history.

Although they depart from a different point of view, this is comparable to the notion of ‘implict self’ from Bomilcar et al. (2021). Building on the work of Damasio, Bomilcar et al. (2021) describe how the self is not only based on conscious knowledge about the self, but in large part also on non-conscious knowledge and non-conscious processes in our brain. These implicit and non-conscious processes influence our responses and can be observed in behavior and attitudes. Although not unequivocally an embodied theory, for the pragmatic purpose this notion of implicit self fits within the understanding of embodied-embedded aspects of self, due to how the outcomes of these non-conscious processes impact behavior.

### Reflective self-aspects

Whereas both the minimal and embodied-embedded aspects of self are primarily pre-reflective, that is preceding explicit, reflective abilities (Butler and Gallagher 2018), the review articles on the self in dementia also delve into eminently reflective abilities.^6^

Reflective abilities (such as memory functioning) are generally perceived as being of vital importance to the self. It’s precisely these abilities that are often impacted by dementia (Hutmacher 2020). Since the self (in dementia) has primarily been understood in terms of reflective abilities, as elaborated on in “From the unitary to the multidimensional self”, section, this understanding still affects present-day research. Even though all of the literature reviews depart from a multidimensional approach (albeit some more far-reaching than others), a clear emphasis on the importance of reflective abilities is still noticeable when it comes to the formulated conceptualizations of self and possible related aspects. On the whole, it delves into the complex relationship between dementia, reflective abilities and the self in various ways, by looking at the (in)ability to update self-knowledge, the distinction between subjective and narrative self-continuity and mental time travel, as described below (Arroyo-Anlló et al. 2020; Baird and Thompson 2018; Bomilcar et al. 2021; Hutmacher 2020; Mentzou et al. 2023; Mograbi et al. 2009; Mograbi et al. 2021; Strickwerda-Brown et al. 2019; Weiler et al. 2016).

An often-mentioned aspect of the reflective self is narrativity (Mograbi et al. 2009; Strickwerda-Brown et al. 2019; Hutmacher 2020). Narrativity is theorized in various ways. However, the starting point in almost all theories on narrativity is that humans are narrative beings. Throughout life humans are constantly actively engaged in the construction of their own narrative (McAdams 2011). This narrative nature is often seen to consist of two intrinsically connected aspects: that human life is constructed in a narrative way and that humans experience themselves narratively (Schechtman 2011). All human lives have a beginning, develop through the choices we make, the experiences we have and the things that happen to us, and ultimately all have an end. In this, every individual has the active leading role in their own life narrative, by shaping their life through decision making, reflecting on what happened in the past and connecting this to present life and to wishes for the future. Narrativity, therefore, isn’t ‘merely having a history’, as Schechtman (2011) writes, but the continual enactment of our own narrative and the continuous rewriting and editing of our personal history (Bohlmeijer et al. 2011). The reflective self, when intact, clearly implicates self-continuity, as reflective abilities, such as memory and self-reflection but also our typical ways of thinking and judging, allow us to understand ourselves as selves, as well as the same selves in different times and places.

In the construction of a life narrative, we often use various reflective abilities to give context to and place our experiences, interactions and thoughts into an overarching story about ourselves. Thinking about oneself as a self requires various types of knowledge about ourselves, in which our memories often play an important role. However, Bomilcar et al. (2021) and Mograbi et al. (2009) address what they view as the inability to update self-knowledge with new memories in dementia. Bomilcar et al. (2021) use the term ‘critical self’ to denote the core of self-identity. Throughout life, humans maintain, what Bomilcar et al. (2021) call, a ‘personal data base’, containing information about ourselves, based on autobiographical memories to which we refer when asked who we are. Usually, this database is continually updated with every new experience we have. However, in the case of dementia, this process of updating is interrupted due to memory impairment. This, consequently, leads to a critical self that is based on remote memories (primarily from early adulthood) and semantic knowledge about the self. Bomilcar et al. (2021) stress that despite difficulty in integrating new experiences into this ‘personal data base’, the critical self can still be seen as the core and continuity of self-identity of PWD, but based on remote autobiographical information.

This view is similar to (and influenced by) the concept of the ‘remote/petrified self’ by Mograbi et al. (2009), in which they focus on Alzheimer’s disease. Mograbi et al. (2009) view the self as comprised by a multitude of brain processes to which we ascribe a sense of unity. This sense of unity follows from ‘a stored representation of individual identity based on personal experience and reconstructive processes’ (Mograbi et al. 2009, p. 992, 998). In this, the self is seen as a ‘commentary instance’, providing interpretation to what we experience by placing our experiences in a bigger picture. Memory, therefore, plays a fundamental role in the ability to maintain and develop a self. However, since Alzheimer’s disease often causes difficulties remembering new information and retrieving memories, Mograbi et al. (2009) argue that the inability to update one’s self with new memories and difficulties with retrieving old memories (‘remote self’) leads persons with Alzheimer's disease to operate from a self that is solidified and based on remote personal semantic information (‘petrified self’) (Mograbi et al. 2009).

Strickwerda-Brown et al. (2019) also focus on the role of memories in their understanding of the narrative self in dementia, but concentrate on the distinction between subjective and narrative self-continuity. They define self-continuity as a ‘sense of self as continuous across time, enabling one to experience both stability and growth in who one is as a person’ (Strickwerda-Brown et al. 2019). According to Strickwerda-Brown et al. (2019), episodic memory provides the basis for *subjective* continuity. By subjective continuity they mean the ability to subjectively relive the past, experience the present and anticipate the future (for example through plans and hopes for the future). This occurs not only through mentally reliving these memories, but also through (re)experiencing the sensory-perceptual details and emotions connected to memories. Semantic memory, on the other hand, provides the basis for *narrative* continuity, by accumulating and storing self-knowledge about personal facts (Strickwerda-Brown et al. 2019). The life story that follows, rich in factual knowledge, emotional depth and personal life themes, can continuously be told and adjusted throughout life, providing a source of meaning and a guide to navigate everyday life by serving a ‘self-schema’ (answers to the questions ‘who I am’, ‘what I do’ and ‘how I behave’). But, in the case of dementia, memory functioning is often impacted in various ways.

Comparable to Mograbi et al. (2009) and Bomilcar et al. (2021), Strickwerda-Brown et al. (2019) argue that in Alzheimer’s disease self-continuity is primarily constituted by narrative continuity, since semantic memories of the remote past are often preserved, leading to an outdated narrative self with little subjective continuity due to a global impairment in episodic memory functioning (including the ability to relive past experiences). According to the authors, this is also the cause of the unawareness of changes in cognitive abilities and personality in persons with Alzheimer’s disease. In semantic dementia self-continuity is constituted primarily by the recent past, since both narrative continuity and subjective continuity are based on semantic and episodic memories of the recent past. This leads to the narrative self of persons with semantic dementia, contrary to the narrative self in persons in Alzheimer’s disease, primarily consisting of recent experiences. In bvFTD, because of the characteristic personality changes, both subjective continuity and narrative continuity are severely impaired. However, with regard to the narrative self of persons living with bvFTD, Strickwerda-Brown et al. (2019) are cautious in drawing conclusions about the implications, since these individuals themselves often don’t perceive their character as being changed.

With regard to self-continuity, both Baird and Thompson (2018) and Arroyo-Anlló et al. (2020) emphasize the importance of re-experiencing the sensorial-perceptual and emotional aspects of memories, similar to Strickwerda-Brown et al. (2019). In their notion of ‘extended self’, Baird and Thompson (2018) refer to ‘the self as it was in the past (according to autobiographical memories) and how we anticipate it to be in the future’ (Baird and Thompson 2018). This doesn’t only imply retrieving and reliving memories, but also the re-experiencing of sensorial-perceptual and emotional aspects of memories. This is also visible in their notion of ‘private self’, understood as ‘conscious experiences that are not available to others such as thoughts, feelings, intentions and dreams’ (Baird and Thompson 2018).

Here they highlight again, illustrated with examples related to music, how sensory input can function as a strong stimulant. Listening to music can evoke memories and therefore be used as a way of supporting the self-continuity of PWD, precisely because music appeals to and elicits emotions and sensorial perceptions attached to memories. For example, listening to a song you used to listen to during the summer when you were 16 can mentally take you back to how that summer was, including reliving sensorial and emotional details, such as the summer heat you felt on your skin, the scent of the flowers in the garden and the happiness you felt during that time. Arroyo-Anlló et al. (2020) demonstrate a similar point in their review on emotional sensorial stimulation in relation to self-consciousness. They understand self-consciousness as the capacity of understanding our own mental world, re-experiencing previous personal events and the ability to interpret thoughts, feelings and beliefs about ourselves, all of which are impaired in Alzheimer’s disease (Arroyo-Anlló et al. 2020). Arroyo-Anlló et al. (2020) argue that sensorial stimulation (such as tastes, music and smells) could help improve memory functioning, because sensorial stimulations invoke emotions, and emotions stimulate remembering.

However, unlike Strickwerda-Brown et al. (2019), in their review focused on Alzheimer’s disease, Weiler et al. (2016), argue that the inability to update self-knowledge might not be the sole complicating factor for self-continuity in PWD. They explicitly mention and highlight the role of mental time travelling. According to them, the self is a representation we form about ourselves, based on factual self-knowledge and our self-narrative. To form such a self-representation, autobiographical memory functioning is required, which consists of the ability of mental time travel and personal semantic memory (Weiler et al. 2016). Mental time travel consists of episodic memory and episodic future thinking. The former is the capacity to re-call and re-experience *past* events. The latter refers to the capacity for imagining possible future scenarios. Mental time travel, then, refers to the ability to transcend the current mental state and occupy several mental states—about the past, present and future—simultaneously. For example, when being nervous about having to do something that scares you in the future, such as giving a presentation for a large audience, thinking about a successful presentation you gave in the past could give you the confidence you need to trust that your upcoming presentation will be a success.

Mental time travel requires complex cognitive abilities, according to Weiler et al. (2016), such as meta-representation (thinking about one’s experiences and thoughts, the experience of subjectivity) and autonoetic awareness (awareness of oneself in time). Again, since persons with Alzheimer's disease often experience impairment in memory functioning—primarily regarding the integration of new memories and retaining recent memories—this, in the eyes of Weiler et al. (2016), leads to the inability to connect memories coherently to each other and place them in a larger narrative. This, consequently, results in a loss of mental time travel abilities, the inability to update the self with new memories, and a narrative self that is primarily constituted by the past. Critically, they argue that it’s not primarily impairment in episodic memory or mental time travel that causes the lack of self-continuity, but impairment in default mode network (DMN) functioning. The DMN refers to certain brain processes that are active when a person is not focused on the external world, and is essential—amongst other mental processes—for updating and integrating older memories with new information from the external world.

### Socially-embedded self-aspects

While the minimal, embodied-embedded and reflective self-aspects primarily depart from the individuals experience, the self can also be understood as predominantly shaped through our interaction with others. Social interactions—i.e. the way we interact with others but also the way in which others interact with us—influence our understanding of our self in implicit and explicit ways. Our understanding of social self-aspects is still strongly influenced by the work of George Herbert Mead. In his work, Mead viewed the self as a continual interaction between, what he called, the ‘I’ and the ‘Me’ (Mead 1913). He defined the ‘I’ as the part of the self that acts without having to think about the action, such as in habits and routines. The ‘Me’ he characterized as the moments when the ‘I’ becomes the object of reflection, that is to say when we think about the actions we undertake. The ‘Me’ should be understood as inherently social, since our reflection on ourselves is shaped by the way others (might) think about us. This can concern a specific person (such as a family member, friend or co-worker), but can also be a ‘generalized other’ (for example a social or cultural view or opinion). Mead emphasizes that this doesn’t only involve opinions and acts that have been expressed to us, but that this also concerns possible opinions we think others ‘might have’ about us that haven’t been articulated (yet) or non-verbal expressions (such as a look of (dis)approval). Mead thus argues that without social interactions and environments, there would be no self, since there would be no ‘Me’. How we think about ourselves throughout our lives is continually shaped and defined by the internalization of how others (might) perceive us.

In this, the close connection between social and embedded aspects of self becomes apparent. Our interactions with our immaterial surroundings—what Mead calls the ‘generalized other’ as mentioned before—such as cultural or social beliefs and norms not only influence our interactions with others, but also the way in which we perceive ourselves. For instance, in some cultures, aging is seen as a process of deterioration and decay, while in other cultures, aging is perceived as gaining wisdom and deserving respect. Depending on which cultural view is the norm where you live, this might not only influence, for example, your interactions with older people or the way you speak about them to others, but also the way in which you perceive your own aging. The social self facilitates self-continuity through the persistence of social roles, interactions and relationships, such as those of a parent, a friend, but also a caring person, a creative person, etc.^7^ The idea that the self is (partly) constituted by our interactions with (specific) others is addressed in a number of the reviews in different ways, such as the role of perspective-taking, personhood as something ‘bestowed’ by others and the importance of social roles, as described below (Bomilcar et al. 2021; Baird and Thompson 2018; Kontos and Martin 2013; Tanner 2013; Tang et al. 2023).

In their notion of the ‘surrogate self’, Bomilcar et al. (2021) focus on the role of perspective-taking. They view perspective-taking as a complex and multifaceted phenomenon that encompasses social and cognitive characteristics and refers to the capacity to take someone else’s point of view. This includes theory of mind (the ability to understand that others have beliefs, desires, intentions, and perspectives of their own, that may differ from ours) and empathy (Bomilcar et al. 2021, p. 6). Bomilcar et al. (2021) argue that by viewing ourselves through the eyes of the other, we can update our own understanding of ourselves by stimulating our self-awareness and ability to reflect on ourselves. According to Bomilcar et al. (2021) perspective-taking is based on the processing of autobiographical ‘self-information’ (episodic and semantic memory functioning), while the processing of ‘other-information’ is primarily based on semantic memory functioning. Regarding the surrogate self in dementia, an individual might experience changes, due to difficulties with memory functioning and therefore perspective-taking. This might lead to a decreased sense of self-awareness, since it could become challenging to view oneself through the eyes of another.

Tanner (2013) approaches the social self in a different way, by departing from Kitwood’s notion of ‘personhood’, defined as: ‘a standing or status bestowed upon one human being by others, in the context of relationship and social being. It implies recognition, respect and trust.’ (Tanner 2013). According to Tanner (2013), the self is intrinsically social and continually shaped throughout life by social interactions with others. Therefore, instead of primarily focusing on cognitive impairment in the understanding of the self in dementia, the focus should be on social interaction and its significance for the ability to maintain a self. The interactions with those surrounding the individual with dementia define the ‘crux of the dementia experience’, since they shape how individuals with dementia perceive and navigate their own sense of self (Tanner 2013).

Tanner (2013), therefore, underscores the importance of recognizing prevalent societal beliefs and stigma related to dementia, such as beliefs that dementia leads to a ‘loss of self’ or results in a ‘zombie-like’ state of ‘living death’ (Tanner 2013, p. 158). These beliefs can influence the understanding of those surrounding the individual with dementia, therefore possibly impacting their way of interacting with the individual with dementia. Since the self, according to Tanner (2013), is largely dependent on these social interactions, this can be of great consequence to the possibilities individuals with dementia have of maintaining their sense of self. For example, people who have the impression that dementia leads to a loss of self might draw the conclusion that visiting their friend with dementia who is unable to recognize them is pointless, because they are ‘no longer there’, while people who believe that, regardless of the changes people might undergo due to their dementia, they are still the same person as they were before would draw the conclusion that visits to their friend are important, even if they are different to the way they used to be. Here, the close connection between social aspects of self and embedded aspects of self becomes apparent, since integrated societal norms and beliefs (embedded self) can be of influence on concrete social interactions (social self). According to Tanner (2013), this can impact the process of ‘self-maintaining’. Self-maintaining is described as the ability to adapt to the changes posed by living with dementia, whilst at the same time also holding on to who one used to be. In this, Tanner (2013) argues that ‘the success of [the efforts of PWD] to manage identity will depend on the support and cooperation of others’, for example by receiving support from others in continuing the shaping of one’s life narrative (Tanner 2013, p. 165).^8^

This close connection between social aspects and embedded aspects of self as presented in the work of Tanner (2013) is also evident in the work of Kontos and Martin (2013) and Bomilcar et al. (2021). Kontos and Martin (2013) argue that our embodied gestures and habits are not only meaningful expressions at the very moment, but they also exhibit the internalization of the social and cultural environment we are a part of, such as class, gender, sexuality and ethnicity. Bomilcar et al. (2021) underline this with their notion of ‘extended self’. With their description of extended self, they argue that the environment, including objects, other people and culture, can be incorporated in the self. This includes interactions with others or cultural influences. Bomilcar et al. (2021) illustrate this with the example of how people diagnosed with dementia in India view their symptoms, where integrated ideas about what is considered normal aging leads people to view their dementia symptoms not as an indicator of an illness, but as a sign of natural aging and therefore no cause for concern or reason to seek medical help (Patel and Prince 2001; Shaji et al. 2002).

The review of Tang et al. (2023) approaches social self-aspects from a different angle, by concentrating on the importance of social roles. By focusing on the dementia experience of persons with ‘young onset dementia’ (people younger than 65 years) they show how our self is largely constructed through the social roles we fulfill. People younger than 65 years often still have a work life and a family with (young) children. These social roles on a work, family and societal level—for example as a co-worker, parent or provider—are under pressure when living with dementia at a young age. Due to these social roles being under pressure, persons with young onset dementia (YOD) encounter difficulties in shaping their self and experience (self-)stigmatization and shame by not being able to fulfill their social roles and thus not being able to live up to social and societal standards. This is often seen in practice, for example, in experiencing shame when not being able to provide financially as ‘provider for the family’ or undergoing stigmatization at the workplace when only being assigned the simplest tasks as a ‘professional’. These pressures concerning social roles result in difficulties shaping their self, since this is primarily based on social interaction with others. According to Tang et al. (2023) persons with YOD try to find the balance between, on the one hand, a need for acceptance regarding their dementia diagnosis, and, on the other hand, a need to be seen for who they were and are. Therefore, Tang et al. (2023) argue that continual engagement in social interactions and activities is crucial since this acknowledges the social being of persons with YOD and provides the possibility to fulfill (new) social roles.

The importance of continued engagement in social interactions and activities is also emphasized by Baird and Thompson (2018) in their understanding of the ‘interpersonal self’ i.e. the self as ‘engaged in social interaction with others’. In their review, Baird and Thompson (2018) emphasize the importance of social interaction with examples related to music. Music is often created or experienced together with others and therefore facilitates the opportunity to strengthen the interpersonal self. They illustrate this, among other examples, with the case study of Betina, a woman with severe Alzheimer’s disease living in a care facility who used to work as a piano teacher. Despite her cognitive impairment she is still able to play the piano and enjoys playing music for others. Through doing so, she establishes a connection with others, thereby preserving part of her social role and a sense of fulfillment within herself.

## Conclusion and discussion

In the coming decades, the rise in people diagnosed with dementia worldwide will confront us with complex challenges, both on a societal and individual level. Not only will the pressure on our healthcare systems increase, which are often already stretched thin in meeting the current demands, informal caregivers (partners, children, family members, friends) will increasingly be tasked with care responsibilities. Ongoing research on how dementia impacts the self—vital for our understanding of what people experience and, therefore, for providing personalized dementia care—however, has resulted in a seemingly disparate theoretical tangle of concepts and frameworks. Therefore, in view of the challenges that lie ahead, the current article aimed to answer the question how the present multiplicity of conceptualizations of self in research on the self of PWD could be integrated and systematized, in view of developing a pragmatic, clustered approach based on the research of the field itself which can be applied in an empirical setting with PWD.

Departing from the literature reviews covering the various and diverse fields involved in the research on the self in dementia, a critical evaluation of the meaning of these seemingly disparate conceptualizations of self in the literature reviews revealed a lot of theoretical overlap. Focusing on the theoretical overlap between these concepts allowed for an inductive formulation of four overarching clusters of self-aspects encompassing all these notions of self: minimal, embodied-embedded, reflective and socially-embedded self-aspects. These clusters can together be seen as providing the ground for self-continuity as a modern, multidimensional interplay of reflective and pre-reflective self-aspects (see also Dings and De Bruin 2022; Gallagher 2013).

This clustered approach is in line with the current developments in research on the self in general, where the shift towards a multidimensional understanding of self shows that these various abilities—like narrativity—should not be understood as restricted to one (specific) aspect of self, but instead as constituted via several aspects of self (at once) in different ways. For example, narrativity—a prime example of an ability which is often almost exclusively understood as a reflective aspect of self—can also be understood in non-reflective terms, for instance as following from embodied expressions like habits as mentioned by Kontos and Martin (2013) and Hutmacher (2020). When certain abilities are impacted by dementia, some self-aspects can become more prominent than others in the constitution and continuity of self.

This clustered approach provides a framework which integrates and systematizes the current field of research and within which new findings can be integrated, rather than continually proposing new taxonomies of the self of PWD. In this, it is important to note that these clusters are already implicitly explored by researchers but not formulated as such. By explicitly formulating them as such, the goal of providing conceptual clarity in and between different research fields, can be facilitated. Not only will conceptual clarity enable the establishment of cross-connections between the various, very different fields involved, it also crosses disciplinary borders and can therefore stimulate interdisciplinary research between research fields and improve the valorization of research findings. For example, Bomilcar’s ‘implicit self’ and Hutmacher’s ‘experiencing self’ may seem different due to their distinct theoretical foundations. However, within this clustered analysis aimed at integrating the conceptualizations of self to contribute to interdisciplinary research intended to improve dementia care, they are functionally very similar. Both terms refer to the pre-reflective, embodied aspects of self.

Nevertheless, this clustered approach also has its limitations. As mentioned, it is a pragmatic notion developed to provide conceptual clarity in the field of research by systematizing and integrating the many unclear and seemingly incompatible conceptualizations. Yet, a critic could argue that the current, non-clustered approach allows space for a more fine-grained, in-depth understanding than this clustered proposal. However, this clustered approach does not imply that these overarching aspects cannot be refined and elaborated on further in other ways. The clustered approach as presented in this article is primarily focused on introducing the general outlines derived from the field itself. However, at the same time, we also need to realize that the aim of ultimately contributing to personalized dementia care that can address all possible care needs following from these different aspects of self, might imply some loss of theoretical nuance to bridge the gap between research and the dementia care practice. Currently, research on the self has resulted in researchers either focusing on one specific aspect of self (often reflective aspects) or—on the opposite side—researchers developing ever more nuanced and fine-grained (sub-)aspects of self, which both have led to the present state of conceptual chaos. Indeed, detailed distinctions help to describe specific phenomena and focused research on specific self-aspects is valuable. However, to integrate the conceptualizations of self and to enhance interdisciplinary research efforts, an approach is needed that navigates between these two opposites. The pragmatic approach presented in this article provides just that: an understanding of how all these various fine-grained and focused notions of self overlap and fit together, thereby offering a workable model useable in empirical research on how PWD experience the impact of dementia on their self themselves.

Consequently, this clustered approach also results in several recommendations for future research. Firstly, a multidimensional approach implies using and developing holistic research methods, encompassing *both* reflective and pre-reflective aspects of self. Research on the self of PWD is often still heavily focused on reflective aspects and abilities. Since dementia can impact these aspects and abilities to a great extent, focusing on these can result in a one-sided, negative view of the self of PWD. A holistic approach corresponds better to the actual lived experience of PWD and provides the opportunity for PWD to express their experience of their self in all its aspects. That is, it would reflect the diverse nature which constitutes the ground for self-continuity. Furthermore, given the disproportionate focus on understanding the reflective aspects of the self in dementia, it is essential to pay particular attention to how the self of people with dementia is expressed through embodied-embedded and socially-embedded means.

Secondly, future research should also become more ecologically valid. Research on the self of PWD is often conducted in a clinical setting, in which people are removed from their familiar (social) environment (for instance, their home or their partner who supports them on a daily basis). In doing so, research runs the risk of portraying a skewed image of the self of PWD, since many aspects of our self are only constituted via (pre-reflective) interaction with our environment and with others. Research conducted in a familiar setting (for example in one’s home or together with others surrounding the PWD) could enable an in depth understanding since it would facilitate these aspects to be expressed and observed. Additionally, theoretical concepts should also be employed in an ecologically valid way. Oftentimes researchers focus on one specific, strictly demarcated notion/aspect of self without taking into consideration the underlying interplay between all the different aspects that constitute the self. A multidimensional understanding of self, however, clearly shows that the various aspects of self have quite diffuse borders and are in dynamic interplay with each other.

Third and last, research on the self of PWD is often conducted primarily via how others (such as partners/family member/loved one/friend etc.) perceive the self of the person with dementia. Future research, however, should focus on developing methods in which the understanding of the self of PWD is less dependent on the interpretation of others surrounding the PWD (not to be confused with the *interaction with* others). As mentioned, people implicitly highlight the importance of reflective abilities and intuitively connect the loss of reflective abilities to the loss of self. This, amongst the internalization of other (implicit) societal beliefs, could influence research results to a great extent. In addition to this, and even more importantly, the literature clearly shows that many expressions of the continuity of self are very subtle, embodied and/or non-verbal. These can therefore only be expressed by the individual themselves and could be hard to recognize and acknowledge by others surrounding the individual. Therefore, future studies on the self of PWD should focus on the PWD themselves.

## Appendix

See Table 2.

**Table 2:** Overview of article aims

	Article	Aim of article
1	Mograbi et al. (2009)	Neuroscientific aim: to establish a link between the self and anosognosia in Alzheimer’s disease, by highlighting the relationship between memory and the self
2	Caddell and Clare (2010)	Critical aim: to create an overview of all types of research methodologies and definitions of self used in qualitative and quantitative research on the self in dementia and, based on this overview, to formulate critique on the disjointed approaches and formulate recommendations for future research on the self in dementia Conceptual aim: to formulate an hypothesis on the impairment of self in dementia based on the reviewed research (self is impacted by dementia, but not lost)
3	Kontos & Martin (2013)	Conceptual aim: criticize the dominant biomedical model of dementia which focusses primarily on cognitive decline Clinical aim: to integrate the notion of embodied selfhood into dementia care practices
4	Tanner (2013)	Conceptual aim: criticize the dominant biomedical model of dementia which focusses primarily on cognitive decline, by highlighting the importance of social processes Clinical/societal aim: improve care practices and awareness of emotional and social needs of persons with dementia in social work
5	Weiler et al. (2016)	Neuroscientific aim: to formulate explanations on where self-awareness deficits originate in Alzheimer’s disease
6	Baird & Thompson (2018)	Clinical aim: to develop a theoretical framework on how music can stimulate all aspects of self by enhancing the feeling of unity and self-continuity, which can be implemented in dementia care practices to contribute to wellbeing
7	Strickwerda-Brown et al. (2019)	Clinical aim: to develop a framework for person-centered care
8	Hutmacher (2020)	Clinical aim: to develop an understanding of the self in dementia primarily based on the experiencing self to inform interactions, interventions and environments
9	Arroyo-Anlló et al. (2020)	Clinical aim: review how the use of sensorial stimuli (odor, music, taste, multi stimuli cues) can be used to invoke autobiographical memories strong in emotional content to contribute to maintaining a sense of self and be a source for promoting wellbeing in Alzheimer’s disease
10	Bomilcar et al. (2021)	Clinical aim: to counter care practices that diminish personhood (‘malignant social psychology’ (Kitwood 1997) by synthesizing research from cognitive neuroscience and social psychology/ethnography on the self and to develop better standards of care based on the Components of the Self Model (CoSM)
11	Mograbi et al. (2021)	Clinical aim: an understanding of changes in self-awareness in dementia patients can contribute to better care practices and counter care practices that neglect personhood (‘malignant social psychology’ (Kitwood 1997))
12	Mentzou et al. (2023)	Clinical aim: to provide a theoretical framework for the development of person-centered psychological care
13	Tang et al. (2023)	‘Lived experience’ aim: to understand what it is like to live with young-onset dementia Clinical aim: need to develop age-appropriate supportive services and care

## Footnotes

1 ‘Integral to our ‘self’’ is not used here in a way to make an implicit ontological claim about what the self ‘is’, it is used to denote the significant impact of dementia on one’s life.

2 The term ‘review articles’ is chosen to refer to articles that synthesize previously published research from their field to provide—among other aims—a comprehensive overview. Some of these articles have additional aims besides providing an overview of the literature and/or integrating the literature, such as developing care strategies or improving awareness around certain dementia related topics. However, the term ‘review article’ is used in the current article to refer to the parts of the review articles used in which they portray the scope of the (current) ongoing research.

3 Thanks to an anonymous reviewer for emphasizing the importance of this point.

4 A diachronic minimal sense of self would have to be based on awareness of the continuity of experiences over time (Fasching 2009). This arguably involves a form of memory or experience-retention that exceeds experience itself, in which the (minimal) self not simply given by the structure of experience.

5 As mentioned in Table 1, next to the ‘proto-self’ and ‘core self’, Damasio’s theory of the self lastly consists of an ‘autobiographical self’. The autobiographical self builds on both the proto-self and minimal self by integrating past experiences and memories into a continuous, reflective sense of identity. As such, while it interacts with minimal self-aspects, it naturally fits with the cluster of reflective self-aspects.

6 Even more so, of all aspects of self, by far most attention, classification and conceptualization occurs within the cluster of reflective self-aspects. Given that various notions fall under the header of reflective self-aspects, it is tempting to provide subclusters for this cluster. However, doing so goes against the purpose of this article, which is to develop overarching conceptual clarity in view of an empirical setting and offer pragmatically useful notions based on the literature itself, rather than theoretically elaborating on every (philosophical) nuance and distinction, or adding subcategories of our own. All these distinctions, are the result of the disproportionate attention that researchers have paid to reflective aspects of the self—something that has not happened with the other aspects. For instance, it would also be possible to distinguish between ‘embodied memory aspects’ and ‘embodied habits aspects’. Therefore, the decision was made to describe the various aspects of reflective self that are mentioned in the review literature, without offering further classification—following the same approach as for the other clusters. When using ‘reflective self’, this refers to the aspect of self that have to do with explicitly thinking about oneself as a self.

7 For an extended analysis, see the work of Tieu and Matthews (2024) and Matthews (2020), who emphasize the importance of understanding selfhood as intersubjective and socially embedded instead of separate from the social and communal environment.

8 Tanner (2013) also delves into the political and moral implications following from her understanding of the social self, by exploring the concept of citizenship. Since this is beyond the scope and focus of the current article, this aspect of Tanners article has been excluded from the analysis.
